# The Pathology and Treatment of Venereal Diseases

**Published:** 1896-04

**Authors:** 


THE PATHOLOGY AND TREATMENT OF VENEREAL DISEASES. By ROBERT W. TAYLOR, A. M., M. D., Clinical Professor of Venereal Diseases in the College of Physicians and Surgeons, New York. In one octavo volume of 1,002 pages, with 230 engravings and seven colored plates. Cloth, $5.50; leather, $6.50. Philadelphia: Lea Brothers & Co., Publishers. 1895.

Within the past few years, many works have appeared upon diseases of the genito-urinary organs, the encyclopedia published under the direction of Pierce Morrow being the most important. We are now presented with another, The pathology and treatment of venereal diseases, by Dr. R. W. Taylor, of New York.

It is not too much to say, concerning this publication, that it is unsurpassed by any of its predecessors, both as regards contents and make-up. Nothing has been omitted and every subject is presented in a concise, lucid, logical manner that cannot fail to impress the student as well as practitioner.

The elimination of old or burdensome matter and an arrangement of facts up to date has been done in the most scholarly manner. Not only has the author given the result of his own experiences, which are enormous, but he has supplemented them by those of others that are worthy of inclusion.

No library should be without this excellent work, and if a selection was limited to but one, this one, it is believed, would be found the most satisfying. Dr. Taylor has added further distinction to himself and placed the profession under many obligations to him. E. W.

